# Efficient screening of pancreatic lipase inhibitors from cod meat hydrolysate through ligand fishing strategy

**DOI:** 10.3389/fnut.2022.969558

**Published:** 2022-08-11

**Authors:** Yongqi Tian, Cuicui Liu, Shaoyun Wang, Ming Du, Beiwei Zhu

**Affiliations:** ^1^School of Food Science and Technology, National Engineering Research Center of Seafood, Dalian Polytechnic University, Dalian, China; ^2^College of Biological Science and Engineering, Fuzhou University, Fuzhou, China

**Keywords:** pancreatic lipase inhibitor, protein hydrolysate, ligand fishing, inhibition mechanism, molecular docking

## Abstract

Obesity has become an increasingly serious public health problem. Pancreatic lipase (PL) is identified as a ideal target for the prevention and treatment of obesity. Orlistat, the only approved PL inhibitor (PLI), is a powerful weight loss drug but has many side effects. Therefore, there is an urgent need to discover powerful PLIs with high safety. Protein hydrolysate has been demonstrated to be a treasure trove of PLIs, but recognizing responsible functional peptides from them is like looking for a needle in a haystack. In this work, we synthesized and optimized a PL ligand fishing model (PLLFM) using magnetic nanoparticles (MNPs), then PLLFM was used to quickly fish out potential PLIs from the Cod meat hydrolysate (CMH). Finally, two new PLIs, GSPPPSG and KLEGDLK were identified with IC_50_ of 0.60 and 1.08 mg/mL, respectively. The Lineweaver-Burk diagram showed that GSPPPSG is a non-competitively dominant mixed-type PLI, whereas KLEGDLK is a competitive inhibitory-type PLI. Moreover, molecular docking suggested that both peptides can stably bind to the key amino acid residues of the PL active site, mainly through hydrogen bonding, hydrophobic, and electrostatic interactions. In general, we not only established a method to rapidly fish out potential PLIs from protein hydrolysate, but also provided safe and efficient lead compounds for the development of novel diet foods or drugs.

## Introduction

Obesity is a state of excessive fat deposition caused by adipocyte hypertrophy and proliferation (1). The latest study showed that in Europe, nearly 59% of adults and 29% of children were either overweight or obese. It is estimated that they cause more than 1.2 million deaths each year in the region (2). Obesity also is considered to be the cause of at least 13 types of cancer, arteriosclerosis, cardiovascular disease, and type 2 diabetes (3, 4). Furthermore, the COVID-19 pandemic has also disproportionately affected overweight people and those living with obesity (5). It is no exaggeration to say that obesity has become one of the main killers threatening human health.

Pancreatic lipase (PL) is a lipolytic enzyme that participates in the hydrolysis of dietary triglycerides for absorption and utilization. It can digest 50–70% of total dietary fat and has been proven to be a crucial target for the development of anti-obesity agents (6). Orlistat is an approved anti-obesity drug with strong pancreatic lipase activity, which can prevent the human body from absorbing about 30% of dietary fat. However, it has serious side effects, such as abdominal pain, flatulence, and oily stool, which often leads to patients' disobedience to treatment (7, 8). Therefore, there is an urgent need to discover powerful PL inhibitors with few side effects. Protein hydrolysates/peptides have attracted extensive attention because of their wide range of biological activities, wide sources, and safety characteristics (9, 10). So far, about 18 PL inhibitory peptides have been screened from millet protein, edible insects, camel milk, pork and chicken skin collagen, and so on (11–15). In addition, PL inhibitory peptides designed and synthesized by pharmacochemical methods have also been reported (16, 17).

How to capture active peptides from complex protein hydrolysates has always been a research hotspot in the field. The traditional activity-guided separation procedures involve repeated fractionation steps and bioactivity tests, which are time-consuming, labor-consuming, and inefficient (18–20). Ligand fishing (LF) is an efficient screening technology developed in recent years, which has been successfully applied to rapidly discover active compounds from complex mixtures (21–23). Affinity ultrafiltration is a typical representative of LF, which has been successfully applied to the screening of XODI and ACEI peptides from protein hydrolysates (23–25). However, the problems of enzyme inactivation, and false-positive in affinity ultrafiltration limit its further application. In order to avoid these problems, magnetic nanoparticles (MNPs) immobilized enzyme technology has been invented, which was highly valued due to its high stability, reusability, and easy separation of ligands (26, 27).

This study aimed to efficient screening of PLIs from protein hydrolysate through ligand fishing strategy. In particular, the pancreatic lipase ligand fishing model (PLLFM) was synthesized and optimized. Then, PLIs were targeted mining, and identification from protein hydrolysate by PLLFM and LC-Q-TOF-MS/MS. Finally, the inhibitory mechanism of active peptides on PL was investigated by Lineweaver–Burk plots and molecular docking.

## Materials and methods

### Materials and reagents

Five protein hydrolysates were from Cod meat, Sea cucumber, *Pseudostellaria heterophylla*, Red snapper scale, and Silver carp skin, and preserved in Dalian Polytechnic University and Fuzhou University. Carboxyl-terminated Fe_3_O_4_ magnetic nanoparticles SM3-P100 (MNPs, 1,150 nm, 10 mg/mL) were purchased from Aorun Weina New Material Technology Co., Ltd. (Shanghai, China). Porcine pancreatic lipase (PL, CAS: 9001-62-1), *p*-nitrophenyl butyrate (*p*NPB, CAS: 2635-84-9) were purchased from Sigma Chemical Co. (St. Louis, MO, USA). *N*-hydroxysucci-nimide (NHS), 2-(*N*-morpholino) ethanesulfonic acid (MES), 1-(3-(dimethylamino)propyl)-3-ethylcarbodiimide hydrochloride (EDC·HCl) were purchased from Macklin Biochemical Co., Ltd. (Shanghai, China). The other chemicals and solvents were all of analytical reagent grade.

### Synthesis and optimization of PLLF model

PLLFM was prepared following a previous paper with a little modification (27). Briefly, MNPs were activated by EDC solution and NHS solution. Then the PL solution was added to a centrifuge tube containing activated MNPs. Finally, the PLLFM were washed 4 times with PBS solution. Through single factor experiments and response surface analysis, the optimal synthesis conditions for the model are determined. Storage stability: PLLFM was stored at 37 and 4°C for 3 weeks. Reusability: PLLFM was washed with PBS and 10% acetonitrile, a total of 7 times.

### Pancreatic lipase inhibition assay

Grind small pieces of cod meat into surimi, add alkaline protease, and hydrolyze for 2 h. The enzyme was then inactivated by high temperature and centrifuged. Finally, the supernatant was spray dried to obtain cod peptide (CP). According to the method of Hou et al. (28), the PL inhibition test was carried out on the protein hydrolysates, its gel chromatography fraction, and the pure peptides. The sample and PL were dissolved in Tris-HCl buffer (pH = 7.5), and *p*NPB was dissolved in acetonitrile, and diluted with tris-HCl buffer. Add 100 μL of the sample, 200 μL of Tris-HCl buffer, 150 μL of PL (5 mg/mL) to a 1.5 mL EP tube, incubate at 37°C for 10 min, and then add 150 μL of 10 mmol/mL *p*NPB, shake and mix, and incubate at 37°C for 10 min. After the reaction was completed, the absorbance value at 405 nm was measured with a multifunctional microplate reader, and the PL inhibitory activity was calculated according to the following equation: Inhibition rate (%) = 1- (absorbance in the presence of inhibitor/fluorescence intensity in negative control) × 100%.

### Fishing PLIs from protein hydrolysates

Five protein Hydrolysates from Cod meat, Sea cucumber, *Pseudostellaria heterophylla*, Red snapper scale, and Silver carp skin were taken to test their LP inhibitory activity. The most active sample was separated by G-15 gel, and the most active fraction was determined as the fishing target. Specifically, 20 μL (10 mg/mL) of the most active fraction (S0) was mixed with the constructed PLLFM and incubated at 37°C for 2 h. After incubation, PBS buffer was added and eluted 3 times to remove non-specific components (S1), and then 10% acetonitrile was used to elute the active ligands with a strong affinity for PL (S2). S0–S2 were analyzed by HPLC (AA12S05-1546WT, 220 nm, 0.5 min/mL), with the mobile phase containing solvent A (0.1%, v/v, of formic acid in water) and solvent B (0.1%, v/v, of formic acid in acetonitrile): 5–55% B at 0–30 min.

### Identification of peptides by nano LC-Q-TOF-MS/MS

The specific peak of S2 was collected and loaded onto Nano LC-Q-TOF-MS/MS (maXis, Bruker Daltonics, Germany) at the Instrumental Analysis Center of Shanghai Jiao Tong University to identify the peptide amino acid sequence.

### Peptide synthesis

The target peptides were synthesized by Cellmano Biotech Co. Ltd. (Hefei, China) by solid-phase method.

### Assay of PLIs kinetics

According to the method reported in the previous literature, with a slight modification, the type of inhibition of PL by active peptides was determined (29, 30). The peptide (0.8 mg/mL, final concentration) was reacted with different concentrations (0, 5, 10, 15, 20 mg/mL) of PL for 10 min, then 10 mM *p*NPB was added for 10 min. the absorbance of the reaction system at a wavelength of 405 nm was measured to determine the reversibility of the reaction. Next, the optimal PL concentration (5 mg/mL) and peptide (0, 0.8 mg/mL, final concentration) were fixed, and *p*NPB with different concentrations (1, 2, 3, 4, and 5 mM) was added to react for 10 min, a value was measured every 30 s, and the wavelength was 405 nm. The Lineweaver-Burk double reciprocal curve was used to determine the maximum velocity (*V*_*max*_) and Michaelis–Menten constant (*K*_*m*_). The slope and *y*-intercept of the Lineweaver-Burk plot vs. peptide concentration give a straight line with the intercepts on the horizontal axis as *Ki* (competitive inhibition constant) and *K'i* values (uncompetitive inhibition constant), respectively.

### Molecular docking analysis

Discovery Studio 2017 R2 software was used to further evaluate the interaction mode and binding affinity between PL and the peptide. The 3D structure of PL (PDB ID: 1LPB) was obtained from the Protein Data Bank database, dehydrated and hydrogenated, and the peptide was minimized by the CHARMM force field. LIBDOCK and CDOCKER were chosen to perform the docking program. Phe 77, Ile 78, Tyr 114, Ser 152, Ala 178, Pro 180, Phe 215, Ala259, and His 263 were active centers (31).

### Statistical analysis

All values in this study were reported as the mean ± standard deviation (SD) based on triplicate independent experiments. Statistical comparisons were performed using Duncan's multiple range test and the least significant difference (LSD) test in SPSS 18.0 software. It was determined to be significant when the *P*-value was below 0.05.

## Results and discussion

### Synthesis and optimization of PLLFM

**Figure 1:**
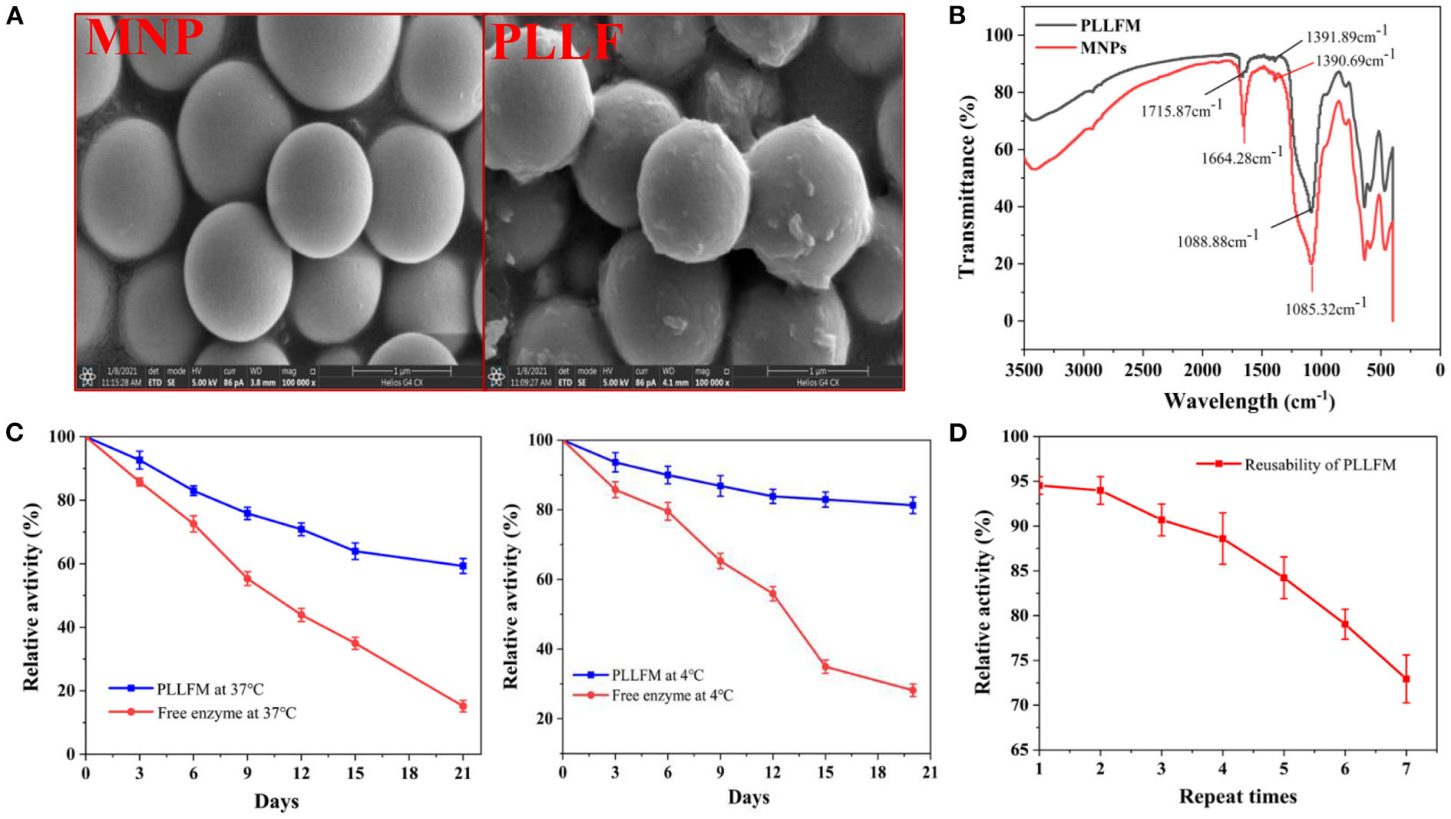
Characterization and properties of PLLFM. **(A)** SEM Characterization. **(B)** FT-IR Characterization. **(C)** Storage stability. **(D)** Reusability of PLLFM.

In order to improve the enzymatic immobilization capacity and relative enzymatic activity of the PLLFM, single-factor experiments and response surface optimization of its synthesis conditions were carried out. The results (Supplementary Figures S1, S2) showed that the optimal immobilization conditions for PLLFM were pH 6.53, enzyme concentration of 25.28 mg/mL, immobilization temperature of 30.69°C, and immobilization time of 3.00 h. Under these conditions, the theoretical enzyme immobilization amount was 144.95 μg/mg, and the relative enzyme activity was 94.27%. The morphology and structure of PLLFM were characterized by scanning electron microscopy (SEM) and Fourier transform infrared spectroscopy (FT-IR), respectively. Compared with MNPs, the surface of PLLFM was rough, fluffy, and had some irregular protrusions (Figure 1A), which means that PL is successfully immobilized on the surface of MNPs. In addition, the FT-IR (Figure 1B) showed that MNPs have a -COOH characteristic absorption peak of the carboxyl group at 1,664.28 cm^−1^, while PLLFM has a broad and strong absorption peak at 1,715.87 cm^−1^, which indicated that -COOH on the MNPs reacts with -NH_2_ on the PL. Subsequently, we examined the stability, and reproducibility of PLLFM. Figure 1C was a comparison chart of the stability of free PL and PLLFM at 37 and 4°C. It can be seen that the longer the storage time, the lower the enzymatic activity. Among them, the enzyme activity of free PL decreased significantly. After 3 weeks of storage, the relative enzymatic activity of PLLFM remained at about 60% (37°C), and 80% (4°C), while the free enzyme activity dropped to about 15 and 20%, respectively. Figure 1D showed the enzyme activity of PLLFM maintained at about 70% after repeated use 7 times. In conclusion, the storage stability and reusability of PLLFM are significantly better than that of free PL, which is consistent with previous literature reports (32, 33).

### Fishing PLIs from protein hydrolysates

**Figure 2:**
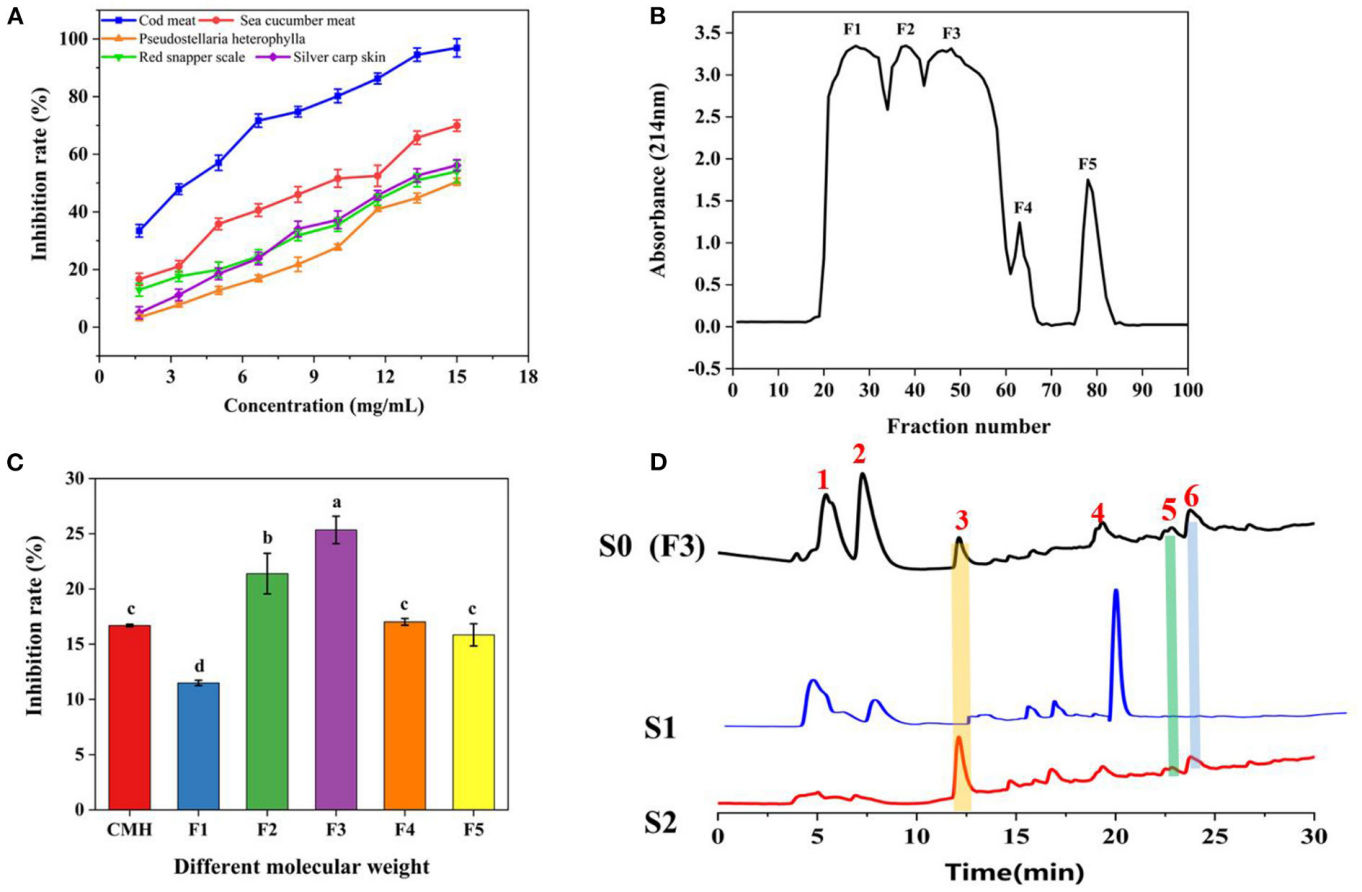
The process of fishing out PLIs from protein hydrolysates. **(A)** Screening of the most active protein hydrolysate. **(B)** Separation of CMH with a gel column G-15. **(C)** Screening of active fractions at a concentration of 1 mg/mL. **(D)** HPLC comparison of active components (F3/S0), non-specific ligands (S1) and specific ligands (S2). The superscript letters indicate significance analysis. The same letters mean no significance.

We used bioinformatics techniques to assist in the screening of protein hydrolysates that may be active. Eighteen reported PL inhibitory peptides were searched from the BIOPEP database, and then the molecular weight distribution, chain length, isoelectric point, net charge, hydrophilic amino acid ratio, etc. were analyzed by online prediction software (34). The result showed that the molecular weight of PL inhibitory peptides was between 800 and 1,500 Da, the length chain was mainly 6–8 amino acids, the isoelectric point was mainly concentrated on 4–7, the net charge was mainly concentrated on −1~0, and the proportion of hydrophilic amino acids was mainly concentrated in 20–50% (Supplementary Figures S3, S4; Supplementary Table S1). By analyzing the commonality of these peptides, we selected 5 qualified protein hydrolysates prepared in our lab, which were obtained from Cod meat, Sea cucumber, *Pseudostellaria heterophylla*, Red snapper scale, and Silver carp skin for PL inhibitory activity tests. As displayed in Figure 2A, the protein hydrolysates all showed a certain PL inhibitory activity, and the Cod meat protein Hydrolysate (CMH) has the best inhibitory effect on PL (IC_50_ = 3.33 mg/mL). Therefore, the CMH was selected as the further research object. Immediately after, the CMH was separated into five fractions (F1-F5) by Sephadex G-15, and their PL inhibitory activities were tested at a concentration of 1 mg/mL. Among them, F3 showed the strongest PL inhibitory ability, and its inhibitory rate was about 1.67 times that of GMH (Figures 2B,C).

F3 (S0) was incubated with PLLFM at 37°C for 2 h, the supernatant was removed by magnetic separation, and eluted with PBS and 10% acetonitrile solution in turn to obtain S1 and S2 eluates for HPLC analysis. As shown in Figure 2D, F3 (S0) detected more than six peaks by HPLC. Peaks 1, 2, and 4 were non-specifically adsorbed species because they were easily eluted by PBS. Peaks 3, 5, and 6 were identified as the targeted ligand because they were only eluted by the denaturant (10% acetonitrile). Among them, peak 3 was collected for further sequence identification due to its large amount, obvious signal, and good water solubility.

### Identification of peptides by nano LC-Q-TOF-MS/MS

**Figure 3:**
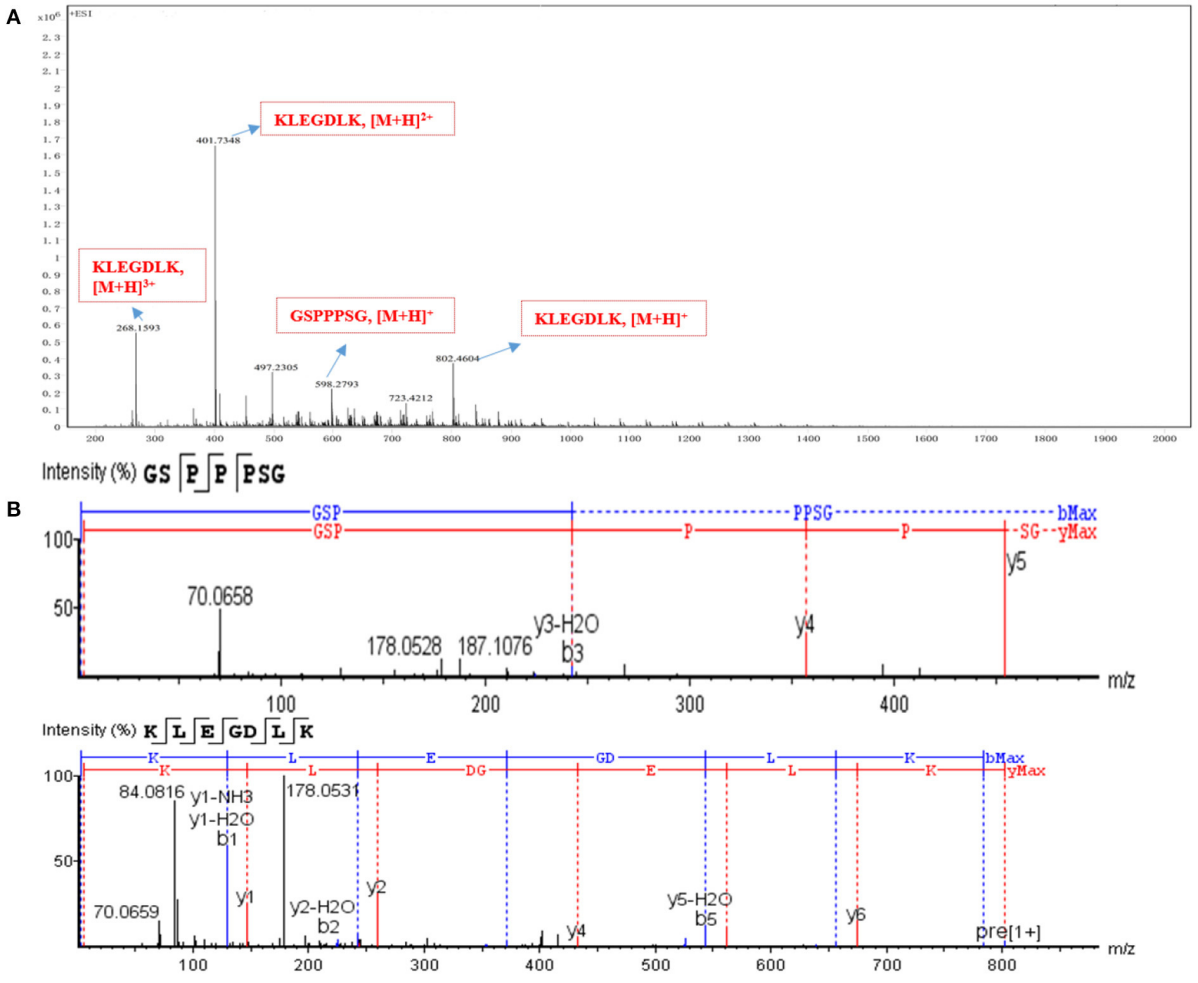
Identification of active peptides. **(A)** The HR-ESI-MS of peak 3. **(B)** the full MS/MS spectrum of GSPPPSG and KLEGDLK.

The characteristic peak 3 was collected, and its sequence was identified by Nano LC-Q-TOF-MS/MS, and further analyzed by Peaks studio 10.0 software combined with the NCBI database. As shown in Figure 3, the two peptides GSPPPSG (m/z 598.2793 [M+H]^+^; −10lgP 34.05) and KLEGDLK (802.4604 [M+H]^+^, 401.7348 [M+H]^2+^, 268.1593 [M]+H]^3+^; −10lgP 48.96), which were considered as potential PLIs for further activity validation due to their high confidence and relative abundance. These two peptides are non-toxic and stable, and their molecular weight, isoelectric point, hydrophilicity and other properties are within the range of the reported physicochemical properties of PL-inhibiting peptides Supplementary Figures S3, S4; Supplementary Table S1).

### IC_50_ and inhibition mode determination of the PLIs activity

**Figure 4:**
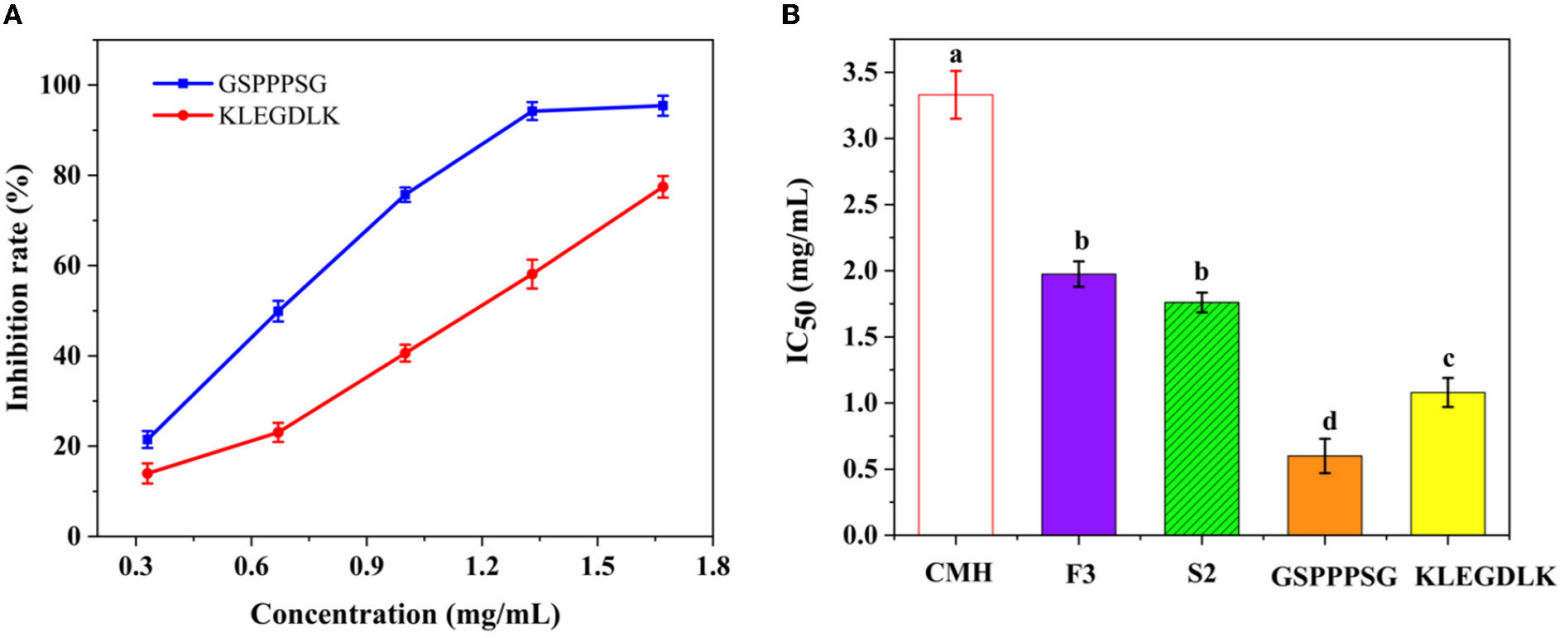
**(A)** The PL inhibitory activity of GSPPPSG and KLEGDLK at concentrations of 0.3, 0.6, and 6.0 mg/mL, respectively. **(B)** the IC_50_ values of CHM, F3, S2, GSPPPSG and KLEGDLK inhibiting PL. The superscript letters indicate significance analysis. The same letters mean no significance.

As shown in Figure 4A, both peptides showed significant inhibition on PL at different concentrations. When the concentration was 6 mg/mL, the residual activity of KLEGDLK on PL was about 10%, while GSPPPSG could almost completely inhibit the inhibition of PL activity. Figure 4B showed the IC_50_ values of different purities of CMH for PL. The IC_50_ value of GSPPPSG for PL was 0.60 mg/mL, and the IC_50_ value of KLEGDLK was 1.08 mg/mL, which were 5.5 and 3.1 times higher than the initial CMH activity (IC_50_ = 3.33 mg/mL), respectively. The activity of these two peptides was about one-third that of orlistat (positive drug, IC_50_ of 14.6 μg/mL), which was also similar to the reported activities of natural products and synthetic compounds (17, 28, 29).

**Figure 5:**
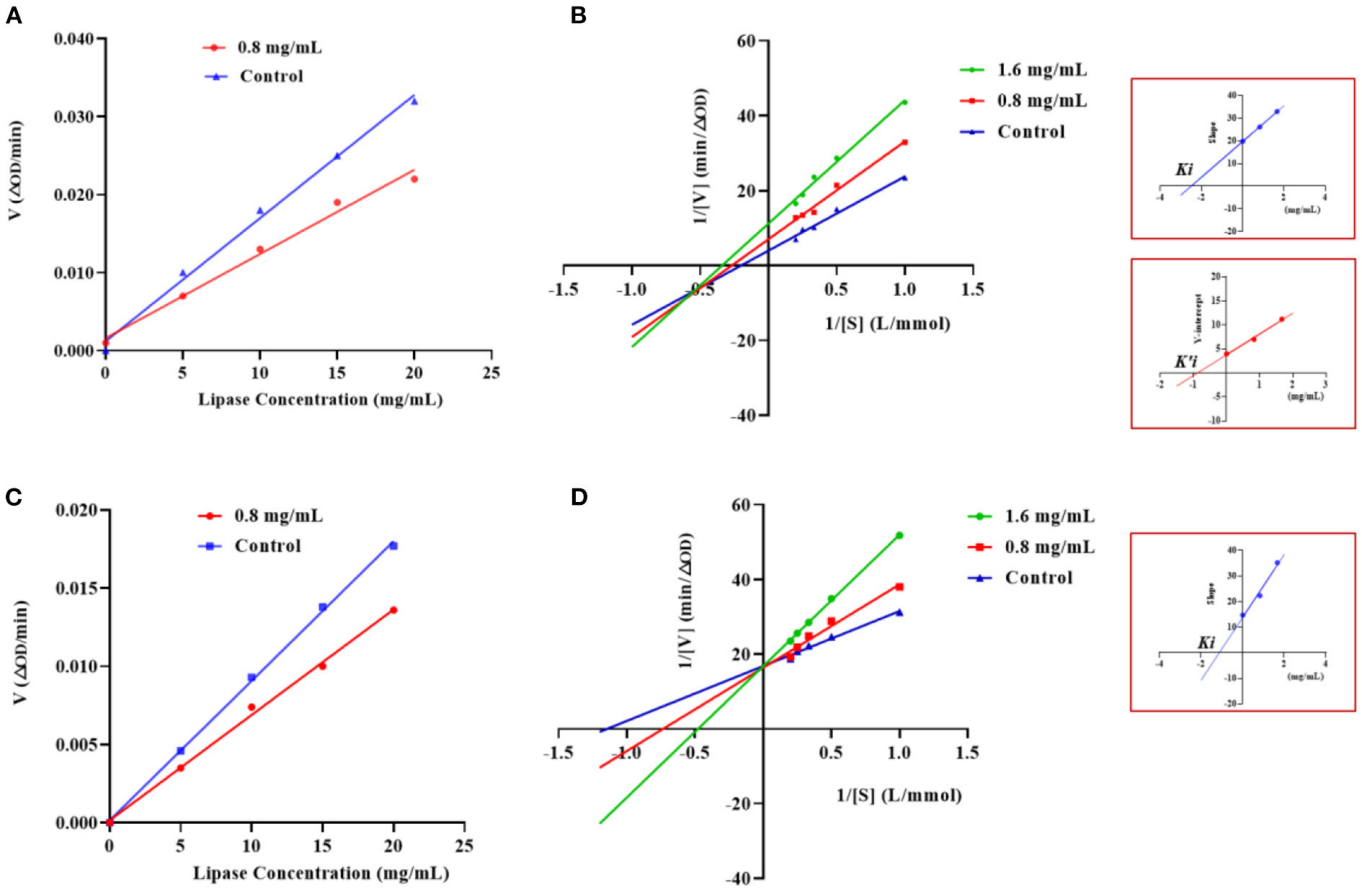
Effects of different concentrations of GSPPPSG **(A)** and KLEGDLK **(C)** on the reaction rate of PL. Lineweaver–Burk plots of GSPPPSG **(B)** and KLEGDLK **(D)**.

The type of inhibition of PL by GSPPPSG and KLEGDLK was shown in Figure 5. Compared to the blank group, the slope of the straight line became smaller after adding GSPPPSG and KLEGDLK, indicating that both peptides are reversible inhibition (Figures 5A,C). Specifically, the straight lines corresponding to different concentrations of GSPPPSG intersect in the third quadrant, and with the increase of concentration, both *K*_*m*_ and *V*_*max*_ gradually decrease (Figure 5B; Table 1). These results suggested that GSPPPSG act as a mixed-type inhibitor of PL, exhibiting both competitive and non-competitive mechanisms. In other words, it competes with the substrate for binding PL and can also bind to the PL-substrate complex. The value of the competitive inhibition constant *K*_*i*_ (4.19 mM) was higher than the non-competitive inhibition constant *K'*_*i*_ (1.46 mM) (Table 1), suggesting that GSPPPSG could bind tightly to the PL-substrate complex (29). Interestingly, KLEGDLK is a competitive PL inhibitor, as judged by an increase in *K*_*m*_ value and a constant *V*_*m*_ value (Figure 5D; Table 1). Furthermore, the *K*_*i*_ value (1.40 mM) of KLEGDLK was lower than that of GSPPPSG, which suggests that KLEGDLK has a stronger affinity for the active site of PL.

**Table 1:** *V*_*max*_, *K*_*m*_, *K*_*i*_, and *K'*_*i*_ of the interaction between PL and GSPPPSG&KLEGDLK.

**Peptide**	**Concentration** **(mg/mL)**	***V_*max*_*** **(ΔOD/min)**	***K_*m*_*** **(mM)**	***K_*i*_*** **(mM)**	***K'_*i*_*** **(mM)**	**Mode of inhibition**
GSPPPSG	0	0.25	4.40	4.19	1.46	Mixed
	0.8	0.14	3.73			
	1.6	0.09	2.95			
KLEGDLK	0	0.06	0.87	1.40	**/**	Competitive
	0.8	0.06	1.32			
	1.6	0.06	2.09			

### Molecular docking analysis

**Figure 6:**
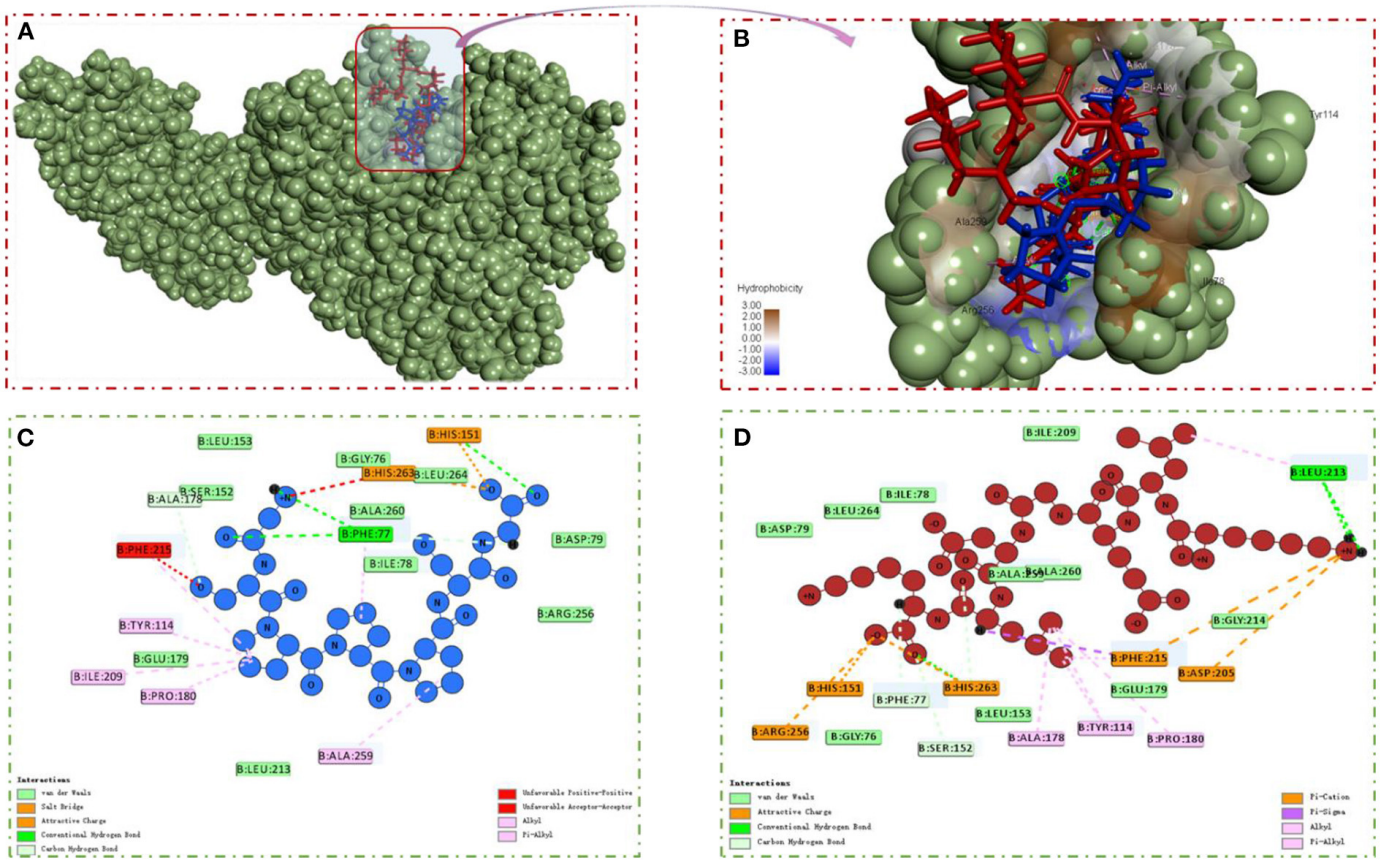
**(A,B)** PL-GSPPPSG&KLEGDLK complex. **(C)** 2D diagram showing interactions between GSPPPSG and PL amino acid residue. **(D)** 2D diagram showing interactions between KLEGDLK and PL amino acid residue.

Molecular docking was performed using the “CDOCKER” and “LIBDOCK” functions in Discovery Studio 2017 R2 software to understand the interaction model of the two peptides with amino acid residues in the PL active pocket (31). From the libdock scores (−57.1333, −141.3610) and cdocked energies (−166.4120, −181.2060 Kal/moL) of GSPPPSG and KLEGDLK, it was inferred that both peptides have high affinity for PL (Table 2). Then, the protein-ligand conformations of these two peptides were analyzed and shown in Figure 6. It can be seen from Figure 6C that six hydrophobic interactions were formed between the prolines in GSPPPSG and amino acid residues of PHE77, TYR114, PRO180, ILE209, PHE2015, and ALA259. Besides, four hydrogen bonds and two electrostatic interactions were also important forces to maintain stability of the PL-GSPPPSG complex (Table 2). The interactions between KLEGDLK and PL were shown in Figure 6D, including five hydrogen bond interactions, five electrostatic interactions, and five hydrophobic interactions (Table 2). The active site of PL (1LPB) is composed of the catalytic triad Ser 152-Asp 176-His 263. This catalytic site is highly restricted by a hydrophobic lid domain consisting of amino acids Gly 76-Lys 80, Leu 213-Met 217 (17, 29, 35). From the above analysis, it can be seen that both peptides can interact with Ser 152 and His 263 of the catalytic center. However, these two peptides, especially KLEGDLK, did not show excellent PL inhibitory activity in the experimental validation, which may be because they lack the group to activate the hydrophobic lid domain, resulting in difficult access to the active site of PL.

**Table 2:** Interaction summary of GSPPPSG, and KLEGDLK with the active site of the PL.

**Compound**	**-CDOCKER ENERGY** **(kcal mol^−1^)**	**-LIBDOCK SCORE**	**Hydrogen bond**	**Charge**	**Hydrophobic**
GSPPPSG	57.1333	166.4120	PHE77, HIS151, ALA178, HIS263	HIS151, HIS263	PHE77, TYR114, PRO180, ILE209, PHE215, ALA259
KLEGDLK	141.3610	181.2060	PHE77, HIS151, SER152, LEU213, HIS263	HIS151, ASP205, PHE215, ARG256, HIS263	TYR114, ALA178, PRO180, LEU213, PHE215

## Conclusion

In summary, rapid fishing of two new PLIs (GSPPPSG, KLEGDLK) from CMH using PLLFM prepared by MNPs was reported for the first time. Peptides GSPPPSG and KLEGDLK showed strong PL inhibition with IC_50_ values of 0.60 and 1.08 mg/mL, respectively. Further studies showed that GSPPPSG is a non-competitively dominant mixed-type PLI, while KLEGDLK is a competitive inhibitory PLI. Molecular docking revealed that both peptides bind to amino acid residues in the active site of PL by hydrogen bonds, hydrophobic interactions, and electrostatic interactions.

## Data availability statement

The original contributions presented in the study are included in the article/Supplementary material, further inquiries can be directed to the corresponding author/s.

## Author contributions

YT conceived, designed research, and wrote the manuscript. CL performed the whole experiment. SW provided the modification of this manuscript. MD and BZ conceived of and proposed the idea. All authors read and approved the manuscript, and agreed to the published the manuscript.

## Funding

This work was supported by National Key R&D Program of China (2018YFD0901106), Natural Science Foundation of China (No. 31771922), and Central Government Subsidy Project (202006).

## Conflict of interest

The authors declare that the research was conducted in the absence of any commercial or financial relationships that could be construed as a potential conflict of interest.

## Publisher's note

All claims expressed in this article are solely those of the authors and do not necessarily represent those of their affiliated organizations, or those of the publisher, the editors and the reviewers. Any product that may be evaluated in this article, or claim that may be made by its manufacturer, is not guaranteed or endorsed by the publisher.
