# Supplementary material for: Efficient screening of pancreatic lipase inhibitors from cod meat hydrolysate through ligand fishing strategy
Source: Front Nutr. 2022 Aug 11;9:969558. doi: 10.3389/fnut.2022.969558 (PMC9403610; doi:10.3389/fnut.2022.969558)
Supplement: Supplementary file 1 [file Data_Sheet_1.PDF]

Efficient Screening of Pancreatic Lipase Inhibitors from Cod Meat Hydrolysate through Ligand Fishing Strategy

Yongqi Tian[1,2], Cuicui Liu[1], Shaoyun Wang[2*], Ming Du[1*], and Beiwei Zhu[1]

[1]*School of Food Science and Technology, National Engineering Research Center of Seafood, Dalian Polytechnic University, Dalian, Liaoning 116034, China*, [2]*College of Biological Science and Engineering, Fuzhou University, Fuzhou, Fujian 350108, China*

*Corresponding author shywang@fzu.edu.cn (S.Y. Wang), duming@dlpu.edu.cn (M. Du)

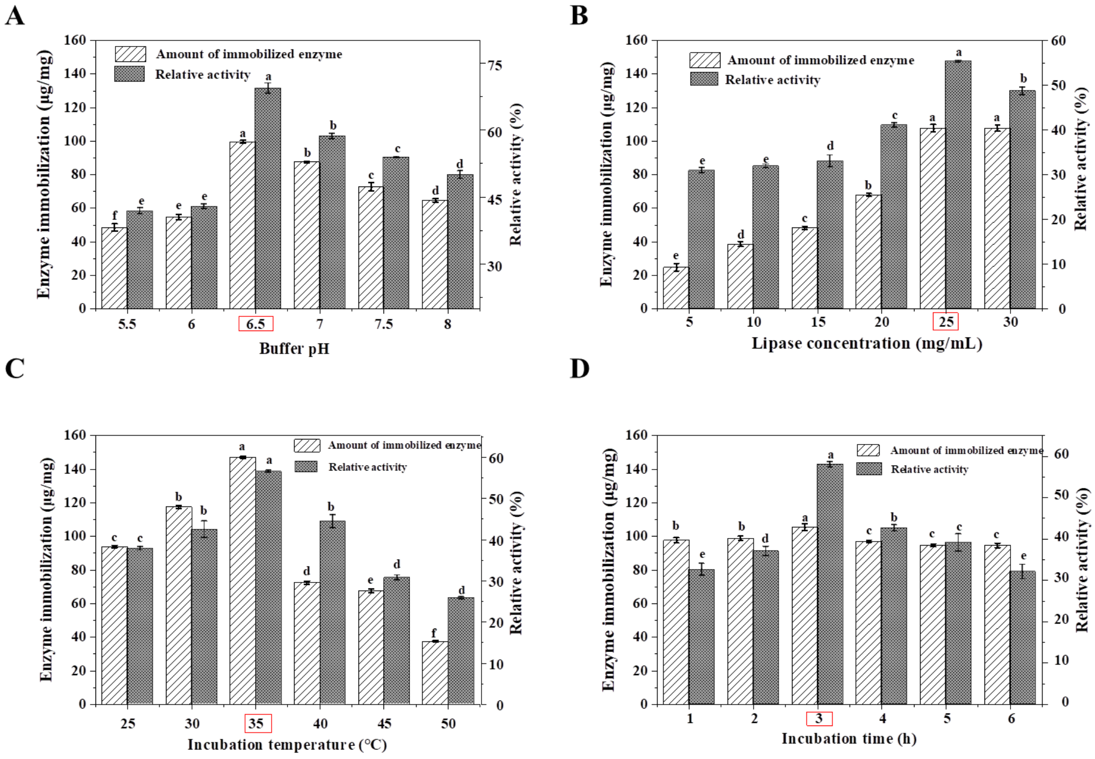

Figure S1 Effects of buffer pH (A), enzyme concentration (B), incubation temperature (C), and incubation time (D) on enzyme immobilizing capacity and enzyme activity of PLLFM.

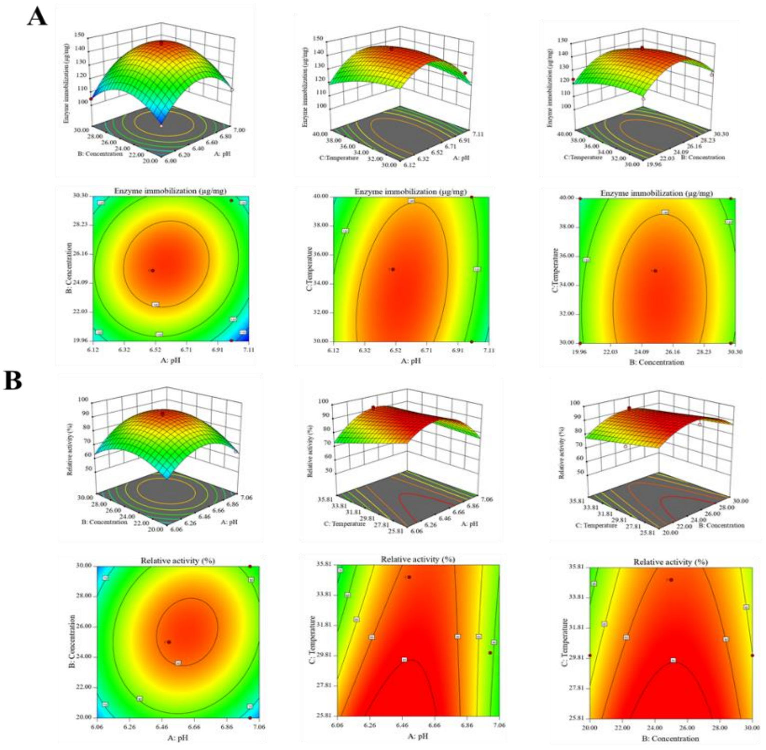

Figure S2 Response surface plots and contour plots of enzyme immobilizing capacity (A), relative enzyme activity (B) under the influence of pH, enzyme concentration and incubation temperature.

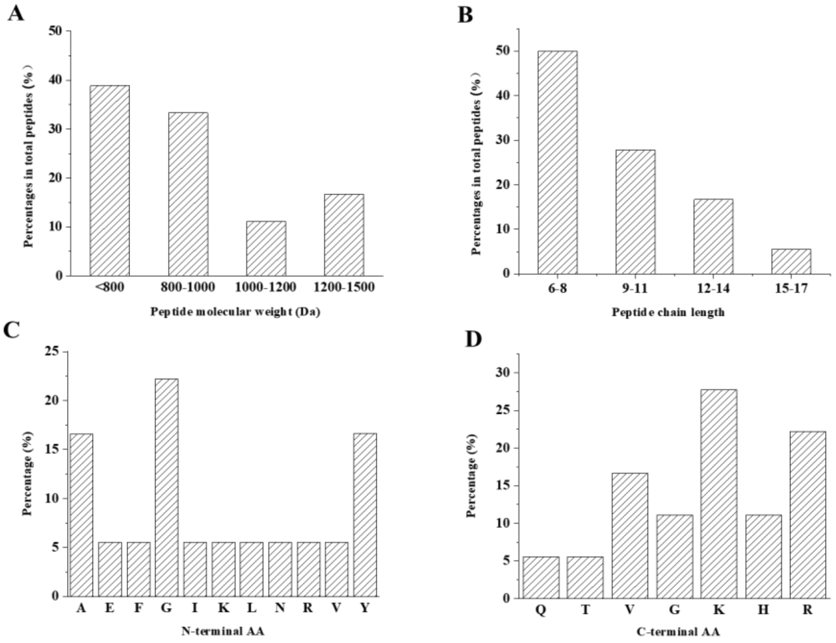

Figure S3 The molecular weight distribution (A), peptide chain length (B) , N-terminal amino acids (C) and C-terminal amino acids (D) of PLIs.

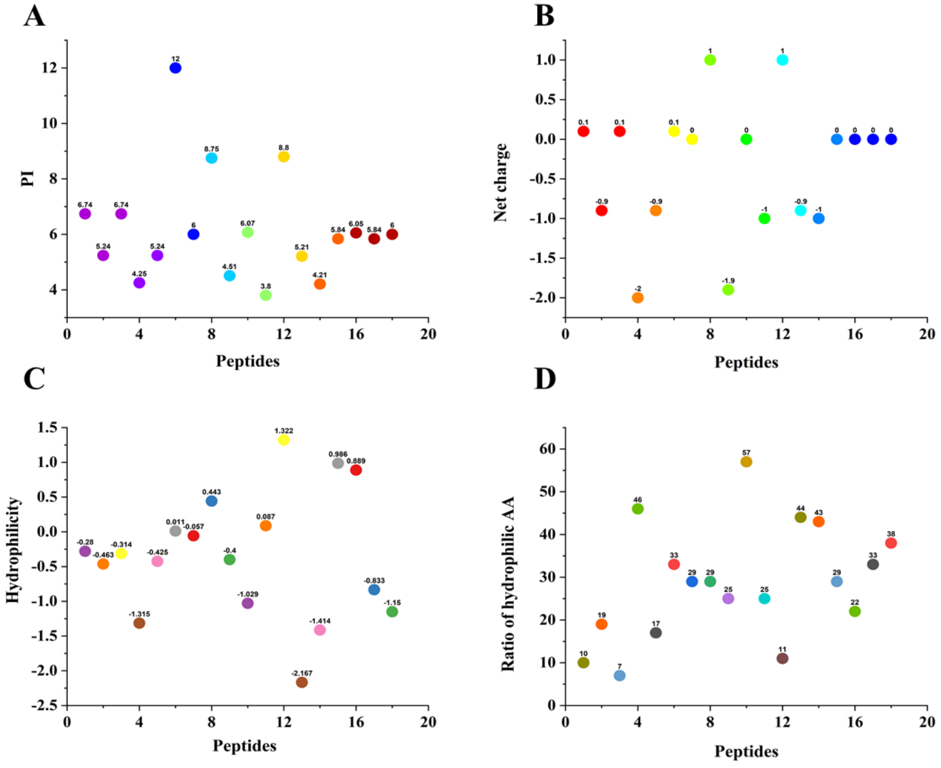

Figure S4 The pI (A), net charge (B), hydrophilicity (C) and the ratio of hydrophilic amino acids (D) of PLIs.

Table S1 physicochemical properties of the PL inhibitory peptides

Ppeptide sequence	Molecular weight	pI	Net charge	Hydrophilicity	Toxicity
YGNPVGGVGH	956.03	6.74	0.1	-0.280	non-toxin
GEHGGAGMGGGQFQPV	1485.59	5.24	-0.9	-0.463	non-toxin
GNPVGGVGHGTTGT	1210.27	6.74	0.1	-0.314	non-toxin
EQGFLPGPEESGR	1402.48	4.25	-2.0	-1.315	non-toxin
GQLGEHGGAGMG	1070.14	5.24	-0.9	-0.425	non-toxin
RLARAGLAQ	955.13	12.00	2.0	0.011	non-toxin
IIAPPER	794.95	6.00	0.0	-0.057	non-toxin
LAPSTIK	728.89	8.75	1.0	0.443	non-toxin
VAPEEHPV	876.96	4.51	-1.9	-0.400	non-toxin
KVEGDLK	787.91	6.07	0.0	-1.029	non-toxin
NYVADGLG	807.86	3.80	-1.0	0.087	non-toxin
AAAPVAVAK	796.97	8.80	1.0	1.322	non-toxin
YDDGSYKPH	1081.11	5.21	-0.9	-2.167	non-toxin
AGDDAPR	700.71	4.21	-1.0	-1.414	non-toxin
GKDAVIV	700.83	5.84	0.0	0.986	non-toxin
AIGVGAIER	885.03	6.05	0.0	0.889	non-toxin
FDPFPK	749.86	5.84	0.0	-0.833	non-toxin
YETGNGIK	880.95	6.00	0.0	-1.150	non-toxin
GSPPPSG	597.63	5.52	0.0	0.1	non-toxin

KLEGDLK	801.94	6.07	0.0	1.2	non-toxin
